# Daily Vinegar Ingestion Improves Depression and Enhances Niacin Metabolism in Overweight Adults: A Randomized Controlled Trial

**DOI:** 10.3390/nu16142305

**Published:** 2024-07-18

**Authors:** Haley Barrong, Hannah Coven, Alexandra Lish, Samantha N. Fessler, Paniz Jasbi, Carol S. Johnston

**Affiliations:** 1 Nutrition Program, College of Health Solutions, Arizona State University, Phoenix, AZ 85004, USA; haley.barrong@asu.edu (H.B.); hncoven@asu.edu (H.C.); arlish@asu.edu (A.L.); samanthanfessler@gmail.com (S.N.F.); 2 Systems Precision Engineering and Advanced Research (SPEAR), Theriome Inc., Phoenix, AZ 85004, USA; jasbi@therio.me

**Keywords:** vinegar, acetic acid, depression, metabolomics, nicotinamide

## Abstract

Depressive disorders are the most prevalent mental health conditions in the world. The commonly prescribed antidepressant medications can have serious side effects, and their efficacy varies widely. Thus, simple, effective adjunct therapies are needed. Vinegar, a fermented acetic acid solution, is emerging as a healthful dietary supplement linked to favorable outcomes for blood glucose management, heart disease risk, and adiposity reduction, and a recent report suggests vinegar may improve symptoms of depression. This randomized controlled study examined the 4-week change in scores for the Center for Epidemiological Studies Depression (CES-D) questionnaire and the Patient Health Questionnaire (PHQ-9) in healthy overweight adults ingesting 2.95 g acetic acid (4 tablespoons vinegar) vs. 0.025 g acetic acid (one vinegar pill) daily. A secondary objective explored possible underlying mechanisms using metabolomics analyses. At week 4, mean CES-D scores fell 26% and 5% for VIN and CON participants respectively, a non-significant difference between groups, and mean PHQ-9 scores fell 42% and 18% for VIN and CON participants (*p* = 0.036). Metabolomics analyses revealed increased nicotinamide concentrations and upregulation of the NAD+ salvage pathway for VIN participants compared to controls, metabolic alterations previously linked to improved mood. Thus, daily vinegar ingestion over four weeks improved self-reported depression symptomology in healthy overweight adults, and enhancements in niacin metabolism may factor into this improvement.

## 1. Introduction

Depressive disorders are the most prevalent and burdensome mental health conditions in the world. Across all ages, the global burden of disease due to depressive disorders increased by 61% between 2009 and 2019 [1]. Additionally, studies show that individuals with depression are pessimistic about treatment efficacy for comorbidities and have worse treatment outcomes as a result [2,3]. Medications and psychotherapy are the standard treatments for depression, and the commonly prescribed antidepressant medications raise the levels of neurotransmitters, most notably serotonin, which conduct the signaling between brain neurons. However, these medications can have serious side effects in some patients, and their efficacy varies widely based on disease severity, comorbidities, and duration of symptoms [4,5]. With this lack of consistency regarding the effectiveness and applicability of medical treatment options for depression, research is warranted to further investigate potential treatment methods. Healthful diet plans and nutritional supplements are gaining recognition as possible therapeutic strategies for preventing and treating depression [6].

Vinegar is a fermented, water-based acetic acid solution that has been a staple in cuisines around the world throughout history [7]. In more recent years, research investigating the role of vinegar as a functional food has increased, and vinegar ingestion has been linked to favorable outcomes for blood glucose management, heart disease risk, and adiposity reduction [7,8,9,10]. In a previous study, we applied metabolomics analyses to serum samples from a randomized controlled 8-week trial to explore metabolic alterations induced by chronic vinegar ingestion and noted a high magnitude of change between groups for the metabolites indole-3-acetic acid and 5-hydroxytryptophan [11]. Furthermore, the functional profile of tryptophan metabolism was differentially expressed [11]. These metabolic shifts suggest enhanced tryptophan metabolism via the indole pathway mediated by the gut microbiota, a shift that would suggest reduced tryptophan degradation through the kynurenine pathway and possibly increased tryptophan availability in the peripheral circulation contributing to increased serotonin production in the brain [12]. In a subsequent controlled clinical investigation, we demonstrated that chronic vinegar ingestion over four weeks significantly improved depressive symptoms in healthy adults, as measured by validated depression screening tools [13].

The defining constituent of vinegars is acetic acid, a short-chain fatty acid (SCFA) that is also produced during carbohydrate fermentation by the gut microbiome. In the intestinal lumen, acetic acid promotes epithelial integrity and reduces many of the inflammatory cytokines that contribute to the depletion of tryptophan via activation of the rate-limiting enzyme, indoleamine 2,3-dioxygenase (IDO), in the kynurenine pathway [14,15]. Acetic acid also promotes the production of a second SCFA, butyrate, by the gut microbiota [16]. Butyrate has a direct role in maintaining systemic tryptophan availability by blocking IDO expression [12,17]. In addition to these potential roles for acetic acid in moderating depressive disorders via enhancement of tryptophan availability, acetic acid converts to acetate once in circulation and is the SCFA with the highest concentration in serum and the brain [18]. In the brain, acetate has been demonstrated to alter neurotransmitter concentrations [19], reduce inflammation [20], and improve hippocampal synaptic plasticity via histone acetylation [21], all linked to favorable brain function.

Since there is a strong mechanistic possibility that dietary acetic acid may influence brain health, and that there is a need to assess the replicability of the antidepressant action of vinegar noted in our earlier trial, we conducted a second randomized controlled trial in healthy, overweight adults to further investigate neurological changes following daily vinegar ingestion for four weeks. The decision to recruit overweight individuals was informed by data from a large, representative U.S. sample (n > 35,000) that demonstrated the lowest risk of depressive symptoms was at BMI < 25.2 kg/m^2^ in males and BMI < 21.1 kg/m^2^ in females [22]. Participants were randomly assigned to ingest either liquid vinegar (2.95 g acetic acid) or a commercial vinegar tablet (0.02 mg acetic acid) daily with meals for four weeks.

The primary objective was to examine the 4-week change in scores for the Center for Epidemiological Studies Depression (CES-D) questionnaire and the Patient Health Questionnaire (PHQ-9) between groups. A secondary objective was to explore possible underlying mechanisms related to the neurological changes using metabolomics analyses. We hypothesized that daily liquid vinegar ingestion would lower depression questionnaire scores after 4 weeks compared to the control treatment (vinegar pill) in a group of generally healthy overweight adults.

## 2. Materials and Methods

### 2.1. Reagents

Reagents included: Methanol (MeOH) (Fisher Scientific, Waltham, MA, USA); Methyl tert-butyl ether (MTBE), O-methyl hydroxylamine hydrochloride (MeOX), N-Methyl-N-(tert-butyldimethylsilyl) trifluoroacetamide (MTBSTFA) and pyridine (Sigma-Aldrich, Saint Louis, MO, USA); Deionized water provided in-house (EMD Millipore, Billerica, MA, USA); Phosphate buffered saline (PBS) (GE Healthcare Life Sciences (Logan, UT, USA); compound standards (Fisher Scientific, Sigma-Aldrich); and Avanti Polar Lipids (Alabaster, AL, USA).

### 2.2. Participants and Study Design

Non-smoking men and women (18–45 years of age; body mass index (BMI): 25–40 kg/m^2^) were recruited from a university community in Phoenix, Arizona using the following inclusion and exclusion criteria: participants were free from chronic disease and/or acute illness, including gastroesophageal reflux disease, frequent heartburn and/or depression diagnosis, were not pregnant or lactating, were not vegetarian or following a weight loss diet, did not report use of recreational drugs, did not consume more than one serving of alcohol daily, and engaged in <75 min of vigorous-intensity physical activity weekly. A score of 1 or higher on question 9 of the PHQ-9 survey (indicating suicidal ideation or other risk for death by suicide) was a disqualifier for study participation, and referrals and hotline contact information was offered. Written informed consent was obtained from all participants prior to study initiation. This trial was approved by the Institutional Review Board at Arizona State University (STUDY00017204) and is registered at clinicaltrials.gov (NCT04706806).

### 2.3. Protocol Procedures

Participants met with study investigators twice at a clinical laboratory facility at baseline and after four weeks. At each visit, participants provided a fasting blood sample, completed a 24-h dietary recall, and had their height, weight, and waist circumference measured by trained study staff. Venipuncture was conducted by a registered nurse on fasting participants (no food or beverages except for water for ≥10 h), with blood collected in serum separator tubes. Tubes were held upright at room temperature for at least 30 min to clot and were centrifuged within 45 min of collection to extract serum (2000× *g* for 10 min at 4 °C), which was frozen (−80 °C) for later metabolomics analyses.

Participants also completed a health history questionnaire and two widely applied, validated depression measures: CES-D and PHQ-9. The CES-D measure is a 20-question Likert scale assessment that assesses how one has felt over the past week with responses ranging from the left ‘rarely or none of the time (less than one day)’ to the right ‘all of the time (5–7 days) [23]. Scores range from 0–60, and scores ≥16 indicate risk for clinical depression [24]. The PHQ-9 measure is a 9-question Likert scale assessment designed for the detection of depression in the primary care setting, with proven reliability and validity for distinguishing the severity of depression [25,26]. Scores range from 0–27; scores of 5, 10, 15, and 20 represent mild, moderate, moderately severe, and severe depression respectively.

To assess recent stressful events as potential confounders, participants completed a 17-item questionnaire that listed occurrences that could impact mood, including significant life events, conflicts with peers, perceived sleep quality on the night prior to assessment, and recent illness [27]. The number of items scored as “yes” indicated the degree of life stress experienced the preceding 3 days. A physical activity score was calculated using the Godin Leisure-Time Exercise questionnaire [28]; scores >24 indicate sufficiently ‘active’.

At the start of the study, participants were provided with the total amount of vinegar supplements needed for the four-week trial. Participants in the active treatment group (VIN) received bottles of red wine vinegar (Pompeian Inc., Baltimore, MD, USA) and were instructed to dilute 30 mL (2 tablespoons) in a cup of water twice daily and ingest at the first bites of a meal. Control group (CON) participants received a bottle of vinegar pills (Spring Valley brand, Walmart.com/Spring Valley) and were instructed to consume one tablet daily at the breakfast meal. The daily dosage of liquid vinegar contained 2.95 g acetic acid; the vinegar capsule contained 22.5 mg acetic acid, an inactive dose [29]. Participants recorded their vinegar intake daily on a study calendar, which was returned to investigators at the end of the trial. In addition, all left over vinegar (bottles or pills) was returned to investigators to assess study compliance (percentage of total dosage ingested over the 4-week study). All vinegar products were commercially purchased and provided to participants in their original containers. Complete blinding to treatment was not possible, but participants were not made aware of whether their supplement was considered the active or control treatment and there was no discussion of an active acetic acid dosage with participants. All participants were instructed not to deviate from their normal diet and physical activity habits during the study.

### 2.4. Targeted Aqueous Profiling with Gas-Chromatography-Mass Spectrometry (GC-MS)

The targeted detection of aqueous metabolites was detailed previously [11,30,31,32]. Briefly, frozen serum samples were thawed under 4 °C. Then, 200 μL 10× diluted PBS and 80 μL of MeOH containing 50 μM PC (17:0, 17:0) and PG (17:0, 17:0) internal standards were added to 20 μL of each thawed sample, and vortexed for 30 s. Afterward, 400 μL of MTBE was added to each sample (MTBE/MeOH/H2O = 10:2:5, *v*/*v*/*v*) and vortexed for 30 s followed by storage in −20 °C for 20 min. Finally, samples were centrifuged at 22,000× *g* at 4 °C for 10 min to separate phases. The aqueous bottom layer (180 μL) from the MTBE extraction was collected into a new Eppendorf tube for derivatization prior to metabolic profiling with GC−MS. The collected bottom layer was dried under vacuum at 37 °C for 4 h using a CentriVap Concentrator (Labconco, Fort Scott, KS, USA). The residues were first derivatized with 40 μL of 20 mg/mL MeOX solution in pyridine under 60 °C for 90 min. Next, 60 μL of MTBSTFA containing d27-mysristic acid were added, and the mixture was incubated at 60 °C for 30 min. The samples were then vortexed for 30 s, followed by centrifugation at 22,000× *g* for 10 min at 4 °C. Finally, 70 μL of supernatant were collected from each sample into new glass vials for GC−MS analysis, while 10 μL was pooled to create a quality control (QC) sample.

GC−MS conditions used here were mainly adopted from previous studies [30,31,32,33]. Briefly, GC−MS experiments were performed on an Agilent 7820A GC-5977B MSD system (Santa Clara, CA, USA) by injecting 2 μL of prepared samples. Helium was used as the carrier gas with a constant flow rate of 1.2 mL/min. The separation of metabolites was achieved using an Agilent HP-5ms capillary column (30 m × 250 μm × 0.25 μm). The column temperature was maintained at 60 °C for 1 min, increased at a rate of 10 °C/min to 325 °C, and then held at this temperature for 10 min. Mass spectral signals were recorded at an m/z range of 60−500, following a 3 min solvent delay. Data extraction was performed using Agilent MassHunter Quantitative Analysis software (version B.07.00). A batch recursive feature extraction algorithm for small molecules was used, and peaks were filtered so that only peaks with absolute height ≥1000 counts were included. The results were integrated using an internal chemical standard library of 126 aqueous metabolites. Following peak integration, metabolites were filtered for reliability and only those with QC coefficient of variation (CV) <20% and relative abundance of 1000 in >80% of samples were retained for analysis. All metabolites were identified with Level 1 confidence according to published criteria [34] using pure chemical standards. Metabolite identification data by GC-MS including concentration and retention time for each metabolite is provided as a supplementary table.

### 2.5. Statistical Analyses

Results are expressed as means ± SD, and all data analyses were conducted using SPSS Version 28.0.1.1 (Statistical Package for the Social Sciences, SPSS, 2012). *p* values were considered significant if ≤0.05. Based on reported effect sizes for supplement trials, averaging 1.1 (range: 0.93–1.3) [35,36], a sample of 30 would provide 80% power to observe a significant change in PHQ-9 scores. To assess differences between groups on baseline characteristics, Mann-Whitney U and chi-squared tests were run for ratio and nominal data, respectively. Univariate analysis was used to assess change in depression scores controlling for confounding variables (age, weight, adherence). A 95% winsorization was applied to change data to limit the impact of outlier data. Spearman’s rho test was used to determine the strength of relationships between variables, and partial eta squared (η^2^) was used to indicate effect size (small, 0.02; medium, 0.13; large, 0.26) [37].

Metabolomics data from serum samples were collected across two analytical batches. Consequently, the ComBat method was used to adjust for batch effects, ensuring the comparability of data across different experimental runs [38]. This statistical approach applies an empirical Bayes framework to stabilize the mean and variance across batches, effectively reducing systematic biases introduced by technical variations (Supplementary Figure S1) [38]. Data were log10-transformed and Pareto scaled (mean-centered and divided by the square root of the standard deviation of each variable) to approximate normality prior to statistical analysis (Supplementary Figure S2). Univariate and multivariate analyses were performed using R and Python languages. Pathway and integrating enzyme enrichment analysis was performed on all captured metabolites and visualized using the MetaboAnalyst 6.0 package [39]. Metabolomic data were mapped to the Kyoto Encyclopedia of Genes and Genomes (KEGG) human pathway library [40], and significance and impact were calculated using a global test of relative-betweenness centrality.

## 3. Results

**Figure 1:**
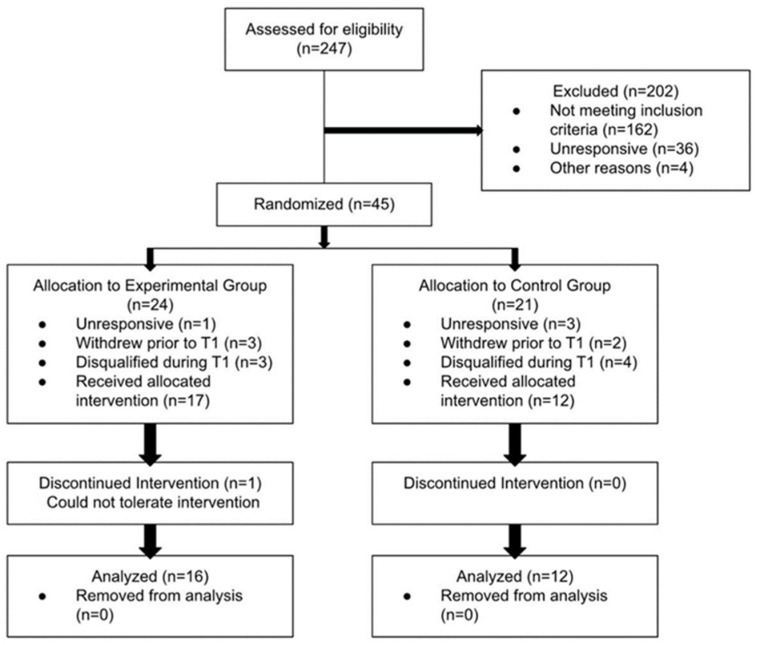
Consort Flowchart.

The study was conducted between January 2023 and June 2023. A total of 247 respondents completed the eligibility screening survey; however, 162 were excluded based on study criteria and 40 were either unresponsive to study team communications or declined to participate (Figure 1). The remaining 45 participants expressed interest in the study; they were stratified by age, sex, and BMI, randomized to the experimental (VIN) group (n = 24) or control (CON) group (n = 21), and scheduled for the baseline visit. Sixteen of these individuals were lost to follow-up or disqualified after further review. Thus, 29 subjects received their supplement allocations and entered the study. One VIN participant did not tolerate vinegar ingestion and withdrew from the study; 28 participants finished the trial. Based on liquid weights and pill counts, adherence to supplement ingestion over the 4-week trial was 90.3% and 101.1% for the VIN and CON groups respectively (*p* = 0.029). Only study completer data were analyzed for this report.

Baseline characteristics did not vary significantly by group (Table 1). A majority of participants were White or Hispanic (50% and 29%, respectively). The mean ages of participants were 25.3 ± 7.3 and 26.3 ± 6.8 years for the VIN and CON groups respectively, and mean BMI ranged from 27.3 ± 3.7 kg/m^2^ to 27.9 ± 3.4 kg/m^2^ respectively. Energy intake did not differ between groups, and 40% of participants took dietary supplements regularly. Medication use was reported by 40% of participants, and two-thirds of these participants were taking a single medication daily. Participants reported similar levels of stressful occurrences in the preceding three days (2–4 events on average), and these numbers did not differ between groups. Physical activity scores did not differ by group, and 75% of participants were sufficiently ‘active’ (scores > 23). Changes in energy intake, physical activity scores, medication or supplement use, and the number of recent stressful occurrences were tracked during the 4-week intervention and did not differ between groups at the end of the trial.

At baseline, the CES-D and PHQ-9 scores did not differ significantly between groups (Table 2). A single participant (VIN group) scored above 15 at baseline on the CES-D measure, indicating possible risk for clinical depression. The PHQ-9 responses at baseline indicated that three VIN participants and one CON participant scored in the range of 5 to 9, indicating mild depression, and one VIN participant (not the same individual who scored high on the CES-D measure) scored in the range of 10 to 14, indicating moderate depression. Thus, 5 participants (18% of the total sample) scored above the threshold indicating a potential risk for depression and mirroring recent national prevalence estimates for depression among U.S. adults: 18.5% [41].

At week 4, the mean CES-D scores fell 26% for VIN participants and 5% for CON participants, and these changes were not significantly different between groups (*p* = 0.544). No participant from either group scored 16 or higher on the CES-D at the end of the trial. The mean PHQ-9 scores fell 42% and 18% for VIN and CON participants respectively, and these 4-week changes differed significantly between groups (*p* = 0.036). Controlling for baseline value raised the *p*-value to 0.059. At trial completion, one participant from each group scored in the range of 5–9, indicating mild depression, representing the highest PHQ-9 scores recorded.

**Figure 2:**
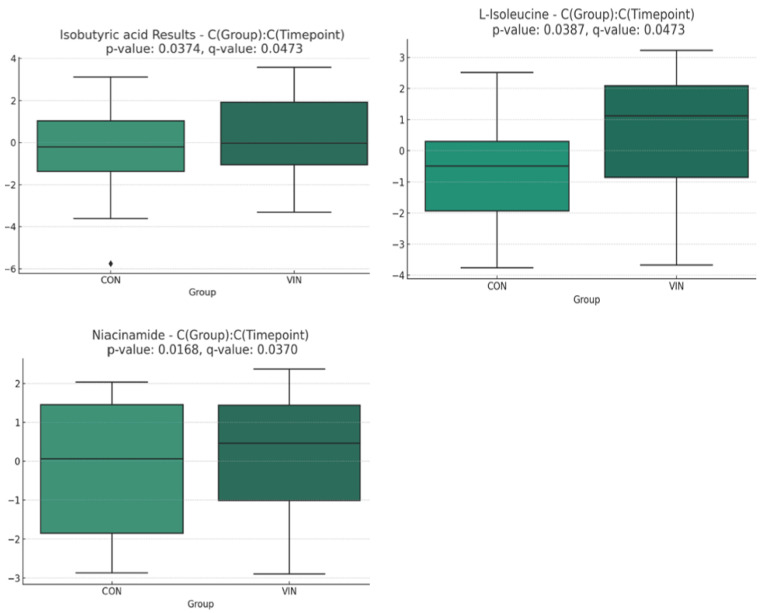
Boxplots for metabolites with significant group x time interactions between VIN and CON participants: isobutyric acid, niacinamide, and L-isoleucine.

**Figure 3:**
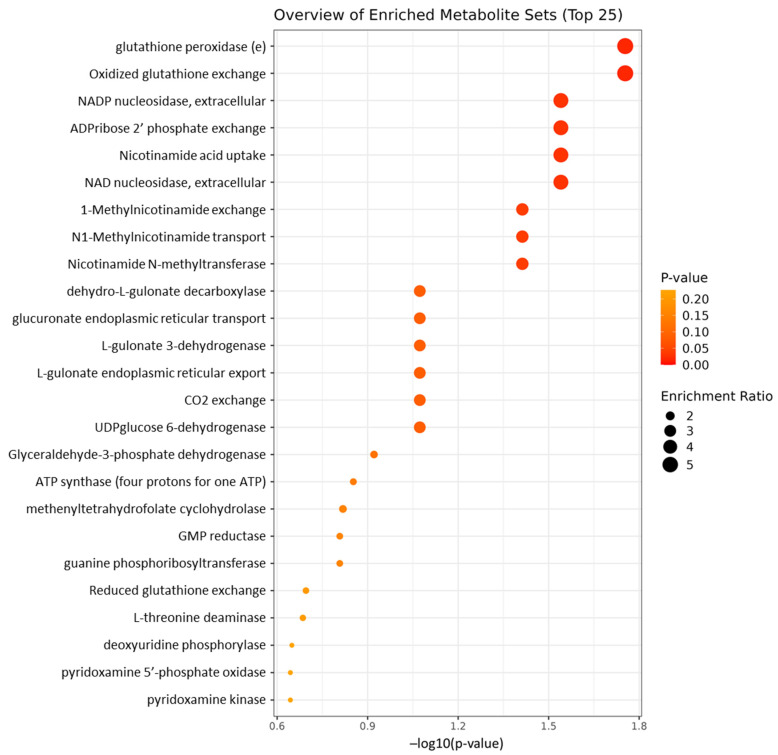
Metabolic pathway enrichment map: the horizontal coordinate is the enrichment significance *p*-value and the vertical coordinate is the KEGG pathway. Displayed are the 25 most important enzymatic pathways differentiating between groups, with colored side bar displaying the relative metabolite concentration in each group. Data analyzed between groups after calculating T2/T1 (post/pre). The first nine pathways listed had significant predicted changes (*p* < 0.05).

Principal components analysis, which included between-group effects, significant differences between timepoints, and group*timepoint interaction effects, identified 11 metabolites with raw *p*-values less than 0.05. After adjusting for multiple comparisons using the False Discovery Rate method (<5%), the significances were maintained. The metabolites that most significantly differentiated the groups over time were isobutyric acid, nicotinamide, glutathione, and L-isoleucine. We recognize that glutathione quantification in plasma is unreliable due to rapid autooxidation during sample collection [42]; moreover, delays in sample processing time increase the amount of glutathione leaking from red cells ex vivo [43]. In the present study, methods to stabilize glutathione and ensure rapid blood processing times were not implemented, and the metabolomic data regarding glutathione are suspect and were omitted (Figure 2). Of the remaining metabolites the raw data revealed that niacinamide and L-isoleucine displayed the greatest change during the trial for VIN participants, +86% and −35% respectively, and these changes were inversely correlated (r = −0.698 [Spearman’s rho]; *p* < 0.001). Enzyme enrichment analysis was performed using all surveyed metabolites, with data analyzed between groups after calculating timepoint2/timepoint1 (post/pre), and nine enzymes were identified with significant predicted changes (Figure 3).

**Figure 4:**
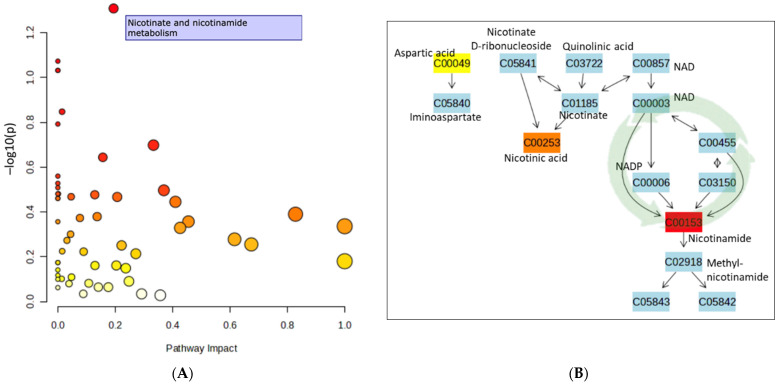
(**A**) PLS-DA Score Plot of Pathway Enrichment Analysis performed using all surveyed metabolites mapped to canonical KEGG pathways. Dots represent change in metabolic activity between groups across time points; size of dot represents the size of the pathway; darker colors (ranging from white to dark red) represent higher hits. (**B**) KEGG IDs referenced in pathway diagram. Key metabolite names are noted. Highlighted boxes represent up-regulated compounds. NAD+ salvage pathway noted with green arrows.

The partial least squares discriminant analysis (PLS-DA) score plot indicated that nicotinamide metabolism was the prominent difference between groups (Figure 4A). The KEGG pathway diagram illustrates the nicotinamide metabolism as inferred from the metabolomics data (Figure 4B). Correlational analyses of baseline data revealed significant inverse relationships for CES-D and PHQ-9 scores and serum niacin concentrations (correlation coefficients ranged from −0.447 to −0.469; *p* < 0.05), supporting the possibility of a link between depression scores and alterations in niacin metabolism.

## 4. Discussion

This study examined the impact of liquid vinegar supplementation on depression scores and the blood metabolome in adults free of chronic disease. To date, little research has been conducted to examine the relationship between vinegar intake and mental health indicators. Self-reported symptoms of depression were quantified using the CES-D and PHQ-9 surveys, which are widely applied, validated screening tools for clinical depression. The change in depression symptoms based on the CES-D survey conducted pre- and post-intervention did not improve significantly with vinegar supplementation compared to the control treatment (−1.63 ± 5.07 and −0.25 ± 5.34, respectively; *p* = 0.544; η^2^ = 0.016). However, PHQ-9 scores did reveal a significant improvement in depression symptoms during the 4-week trial in the VIN participants in comparison to control (−1.31 ± 2.18 and −0.33 ± 0.98, respectively; *p* = 0.036, η^2^ = 0.178), but when the baseline score was added as a covariate in the analysis, the *p*-value rose above 0.05 (*p* = 0.059, η^2^ = 0.152). 

In our previous randomized controlled study, a significant reduction in CES-D scores (−34%) was noted with vinegar ingestion in comparison to the control after 4 weeks [13]. The previous study was conducted during the COVID-19 lockdown and baseline CES-D scores for the treatment group were 2-fold higher than the present study (13 vs. 6), and participants with scores ≥ 16 (suggestive of risk for clinical depression) were also greater: 5 (36%) vs. 1 (6%) [13]. These data offer the possibility that the efficacy of the vinegar treatment could improve in groups with worse baseline mental health scores. Furthermore, it is interesting to note in the present study that, at baseline, 25% of VIN participants tested at mild or moderate depression according to the PHQ-9. This could indicate that the PHQ-9 may have increased sensitivity to symptomology, and therefore a similar potential sensitivity to intervention effects. Additionally, according to the most common definitions for ‘clinical symptom response’ in the psychology field, there is a treatment response at 50% change on a valid symptom rating scale and a partial response at 25% change [44,45]. Accordingly, the score changes seen in our study show a partial response in the VIN group in both surveys (25.8% change for CES-D and 42.8% for PHQ-9) compared to no response in the CON group (5.1% for CES-D and 18.9% for PHQ-9).

Previous research has demonstrated that behavioral strategies for depression treatment may be sufficient to illicit symptom change, irrespective of other interventions [46]. This idea could be extrapolated to hypothesize that the more behavioral changes implemented, or more of the same positive behavioral change, could further improve depression symptoms. With VIN participants being required to participate in habitual behaviors twice as often as CON participants (liquid vinegar ingestion twice per day with meals compared to a single pill consumption daily), the role of behavioral change is another factor to be considered when interpreting these results. Moreover, red wine vinegar is known to have some of the highest polyphenol contents among fruit vinegars, and the antioxidant effects of these compounds should be considered when discussing the noted changes to depression scores in this study [47]. Red wine vinegar retains comparable antioxidant properties compared to whole black grapes but has much lower antioxidant capacity than red wines [48]. Recent research has shown inverse associations between the consumption of high-polyphenol beverages (such as red wine, tea, and coffee), stress, and depression symptomology [49].

Metabolomics analyses indicated that acetic acid ingestion affects the metabolism of nicotinamide, isoleucine, and isobutyric acid since their loadings effectively distinguished between the treatment and control groups over the course of the study. Analyses of the raw data showed significant concentration changes (study week 4 minus baseline) for the three metabolites: nicotinamide (+86% vs. +5%), isoleucine (−35% vs. −5%), and isobutyric acid (−3% vs. +31%) for VIN and CON respectively (*p* ≤ 0.05). There is supporting evidence in the literature linking two of these metabolites to depressive symptoms. Liu et al. demonstrated that nicotinamide ingestion, dissolved in drinking water at a 200 mg/kg/day dose, fully reversed depressive symptoms in mice exposed to restrain stress [50]. Using non-targeted metabolomics, Chen et al. identified isobutyric acid as one of two metabolites linked directly to bipolar disorder in patients (n = 55) vs. healthy adults (n = 110) [51]. A recent report, however, was unable to link isoleucine to depressive symptoms. Utilizing a longitudinal Finnish population-based cohort (n = 725), Whipp et al. applied metabolomic analyses to investigate links between metabolites, with a specific focus on the branched chain amino acids, and depression rankings. Isoleucine was not associated with depressive symptoms in this cohort; however, the other branch chain amino acids, leucine and valine, did register significant negative associations with depressive symptoms [52].

Based on the patterns of metabolite concentration changes between the VIN and CON groups over time, the MetaboAnalyst software 6.0 generated a KEGG pathway (Figure 4B) that begins at a midpoint of the kynurenine pathway of tryptophan metabolism (e.g., quinolinic acid) to produce NAD+. NAD+ is present in all tissue cells and participates in cellular energy production as well as numerous oxidation-reduction reactions and cellular signaling. When NAD+ is consumed, nicotinamide and adenosine diphosphate ribose are generated, a reaction catalyzed by NAD nucleosidases, metabolite sets noted in the enrichment analyses herein. The conversion of NAD+ to nicotinamide activates sirtuins (e.g., sirtuin-1) and poly[ADP-ribose] polymerases (PARPs), which promote mitochondrial biogenesis, energy expenditure, and antioxidant defenses [50,53] and DNA repair [54], respectively. In the brain, mitochondrial quality is linked with reduced risk of neurodegenerative diseases, and emerging evidence suggests that sirtuins play a key role in orchestrating this balanced mitochondrial fusion and fission [55,56,57]. Moreover, the PARPs protect neurons from the oxidative stress commonly generated in vivo during metabolic activities [54,58]. Nicotinamide is readily converted back to NAD+ in a recycling system known as the salvage pathway. Since the cellular requirement for the NAD+ cofactor is greater than that which can be endogenously generated, this salvage pathway has critical significance for cellular energetics and neuroprotection [59,60].

Thus, it is plausible that acetic acid ingestion enhances the NAD+ salvage pathway, elevates sirtuin and PARP activity, and promotes mitochondrial biogenesis and cell integrity in the brain. Others have demonstrated that increases in nicotinamide, via the nicotinamide salvage pathway, were associated with improved depression symptomology in humans, independent of other neuroactive metabolic pathways, and shown to ameliorate depressive symptoms in animals [50,61,62]. In our previous investigation, the first controlled vinegar intervention trial employing metabolomic analyses, the pathway and enrichment analyses found tryptophan metabolism to be differentially expressed in response to daily vinegar intake [11]. However, those analyses suggested an increased tryptophan flow through the indole pathway, not the kynurenine pathway, as indicated by a high magnitude of change between groups for the metabolites indole-3-acetic acid and 5-hydroxytryptophan. These conflicting metabolomic results warrant further investigation in future intervention studies.

**Figure 5:**
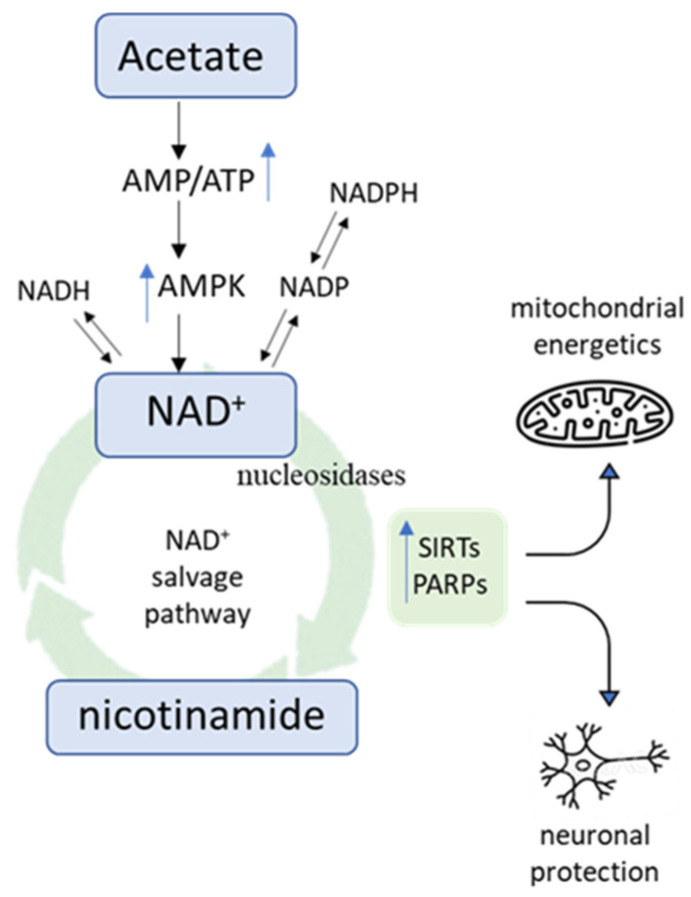
Acetate induced AMPK signaling to promote NAD+ cycling to nicotinamide. Activation of the SIRTS and PARPs occurs which function in mitochondrial energetics and neuronal protection.

There is the possibility that the metabolomic data presented in the present report could be interpreted to support an alternate metabolic system: AMP-activated protein kinase (AMPK) signaling to increase NAD+ metabolism and SIRT1 activity [63,64]. Moreover, the involvement of AMPK signaling in NAD+ metabolism and its salvage pathway is well documented [64,65,66]. In vivo, Yamashita et al. demonstrated that acetate administration increased the phosphorylation of AMPK [67]. In vitro, acetic acid administration was demonstrated to activate AMPK via conversion to acetyl-CoA with the concomitant rise in AMP [68,69]. Further research is needed to examine whether vinegar ingestion stimulates AMPK signaling and links to improvement in mood state (Figure 5).

Although metabolomic analyses are being applied to biological samples with rapidly increasing frequency in the past decade, the ability to process and interpret the data is considered ‘severely limited’ and generally focused on only a few metabolites [70]. The tenuous connection between the metabolomic analyses for two independent vinegar supplementation trials in healthy adults (e.g., alterations in tryptophan metabolism in response to vinegar intake) is encouraging, but further investigations are needed to clarify these metabolic changes and directly link them to depressive symptoms. Data interpretation is also limited by the small sample size. Future vinegar supplementation trials in adults with clinical depression would also clarify a role for acetic acid ingestion in moderating depressive symptoms.

## 5. Conclusions

These data provide additional support that daily vinegar ingestion over four weeks can improve self-reported depression symptomology in generally healthy adults and that alterations in niacin metabolism may factor into this improvement. Future research examining the effects of vinegar administration in clinically depressed or at-risk populations, and those on antidepressant medications, is warranted. A focus on mechanisms and large patient samples will strengthen the science and provide the evidence to more firmly demonstrate vinegar’s role in health promotion. Vinegar is inexpensive, easily incorporated into diet, and widely accessible. The culinary history of vinegar dates back thousands of years across many cultures; adding vinegar to sauces, dressings, and marinades may achieve a much greater benefit than simply spicing up the diet.

## Figures and Tables

**Table 1:** Participant characteristics at baseline by group assignment: VIN (liquid vinegar) and CON (vinegar tablet).

	VIN	CON	*p* ^a^
Sex, n			
Male/Female	4/12	3/9	0.666
Age, y	25.3 ± 7.3	26.3 ± 6.8	0.423
Body mass index, kg/m^2^	27.3 ± 3.7	27.9 ± 3.4	0.450
Weight, kg	79.0 ± 15.2	79.9 ± 13.1	0.732
Race/Ethnicity, n (%)			
White	9 (56.3)	6 (50.0)	0.521
Black and African American	2 (12.5)	2 (16.7)	
Native American	0 (0.0)	1 (8.3)	
Hispanic	5 (31.3)	3 (25.0)	
Education, n (%)			
High school diploma	6 (37.5)	4 (33.3)	0.521
AA/vocational degree	1 (6.3)	2 (16.7)	
College degree	6 (37.5)	4 (33.3)	
MS degree	3 (18.8)	2 (16.7)	
Energy intake, kcal	2189 ± 566	2166 ± 838	0.664
Alcohol Intake, servings/day	0.44 ± 0.60	1.21 ± 2.87	0.945
Supplement use, Yes/No	6/10	5/7	0.823
Medications, dosages/day	0.69 ± 1.08	0.50 ± 0.67	0.945
Stress Score, n	3.88 ± 2.45	2.42 ± 2.31	0.159
Physical Activity score	35.6 ± 17.0	38.3 ± 20.4	0.767

^a^ *p* values represent the Mann-Whitney U test and chi square test for ratio and nominal data, respectively.

**Table 2:** CES-D and PHQ-9 Scores at Baseline and at Week 4 for VIN and CON participants^.^

	VIN	CON	*P* ^a^	η^2^
CES-D				
Baseline	6.3 ± 5.4	4.92 ± 5.0		
Week 4	4.7 ± 3.3	4.7 ± 3.3		
∆	−1.63 ± 5.07	−0.25 ± 5.34	0.544	0.016
PHQ-9				
Baseline	3.1 ± 3.1	1.8 ± 2.0		
Week 4	1.8 ± 1.9	1.4 ± 1.9		
∆	−1.31 ± 2.18	−0.33 ± 0.98	0.036	0.178

^a^ *p* values represent Univariate analysis controlling for confounding variables (age, weight, adherence). η^2^ = effect size. 95% winsorization was applied to change data to limit the impact of outlier data.

## Data Availability

The data presented in this study are available upon request from the corresponding author.

## Supplementary Materials

The following supporting information can be downloaded at: https://www.mdpi.com/article/10.3390/nu16142305/s1. Figure S1: A. Principal Component Analysis (PCA) showing general profile before and after correction. B. Singular Value Decomposition (SVD) analysis applied to find systematic trends attributable to bias; Figure S2: (A). Before any normalization: Shows the original data distributions. (B). After log transformation: Illustrates the effect of log transformation on the data, aiming to reduce skewness and scale the values. (C). After both log transformation and Pareto scaling: Demonstrates the distributions after applying both preprocessing steps, which further modifies the data to potentially enhance statistical analysis or model performance. Supplementary Table: Metabolite Identification Data by GC-MS.
