# Supplementary material for: Daily Vinegar Ingestion Improves Depression and Enhances Niacin Metabolism in Overweight Adults: A Randomized Controlled Trial
Source: Nutrients. 2024 Jul 18;16(14):2305. doi: 10.3390/nu16142305 (PMC11280469; doi:10.3390/nu16142305)
Supplement: Supplementary file 1 [file nutrients-16-02305-s001.zip › nutrients-3066347-supplementary.pdf]

Supplementary Table: Metabolite Identification Data by GC-MS

HMDB ID	Metabolite Name*	Derivative	Retention Time (sec)	Retention Time (min)	Base Peak (m/z)**
HMDB0000002	1,3-Diaminopropane	4 TMS	731.678	12.195	174
HMDB0000008	2-Hydroxybutyric acid	2 TMS	284.814	4.747	73
HMDB0000020	p-Hydroxyphenylacetic acid	2 TMS	831.863	13.864	179
HMDB0000034	Adenine	2 TMS	969.562	16.159	264
HMDB0000050	Adenosine	4 TMS	1190.610	19.844	236
HMDB0000056	beta-Alanine	3 TMS	1441.000	24.017	174
HMDB0000058	Cyclic AMP	3 TMS	660.268	11.004	73
HMDB0000072	cis-Aconitic acid	3 TMS	851.612	14.194	147
HMDB0000076	Dihydrouracil	2 TMS	292.351	4.873	73
HMDB0000094	Citric acid	4 TMS	859.263	14.321	147
HMDB0000099	L-Cystathionine	4 TMS	519.952	8.666	73
HMDB0000101	Deoxyadenosine	3 TMS	1208.060	20.134	207
HMDB0000112	gamma-Aminobutyric acid	3 TMS	716.713	11.945	174
HMDB0000115	Glycolic acid	2 TMS	266.632	4.444	73
HMDB0000123	Glycine	3 TMS	582.463	9.708	174
HMDB0000124	Fructose 6-phosphate	6 TMS	1029.360	17.156	315
HMDB0000125	Glutathione	x TMS	789.613	13.160	156
HMDB0000131	Glycerol	3 TMS	541.409	9.023	147
HMDB0000133	Guanosine	5 TMS	1226.810	20.447	103
HMDB0000134	Fumaric acid	2 TMS	635.212	10.587	245
HMDB0000148	L-Glutamic acid	3 TMS	779.962	12.999	246
HMDB0000149	Ethanolamine	3 TMS	529.512	8.825	174
HMDB0000156	Malic acid	3 TMS	715.279	11.921	147
HMDB0000157	Hypoxanthine	2 TMS	946.263	15.771	265
HMDB0000158	L-Tyrosine	3 TMS	931.512	15.525	218
HMDB0000159	L-Phenylalanine	2 TMS	813.128	13.552	192
HMDB0000161	L-Alanine	1 TMS	425.663	7.094	116
HMDB0000162	L-Proline	2 TMS	1459.900	24.332	142
HMDB0000167	L-Threonine	3 TMS	629.063	10.484	117
HMDB0000168	L-Asparagine	3 TMS	827.663	13.794	116
HMDB0000169	D-Mannose	5 TMS	842.162	14.036	147

HMDB0000172	L-Isoleucine	2 TMS	580.209	9.670	158
HMDB0000177	L-Histidine	3 TMS	962.513	16.042	154
HMDB0000181	L-Dopa	4 TMS	973.513	16.225	218
HMDB0000182	L-Lysine	3 TMS	884.312	14.739	174
HMDB0000186	Alpha-Lactose	8 TMS	1112.810	18.547	204
HMDB0000187	L-Serine	3 TMS	620.462	10.341	204
HMDB0000191	L-Aspartic acid	3 TMS	730.212	12.170	232
HMDB0000192	L-Cystine	4 TMS	1061.260	17.688	218
HMDB0000193	Isocitric acid	4 TMS	870.763	14.513	147
HMDB0000195	Inosine	4 TMS	613.296	10.222	73
HMDB0000197	Indoleacetic acid	2 TMS	1016.440	16.941	202
HMDB0000202	Methylmalonic acid	2 TMS	566.759	9.446	147
HMDB0000205	Phenylpyruvic acid	1 TMS	851.878	14.198	89
HMDB0000207	Oleic acid	1 TMS	555.547	9.259	73
HMDB0000208	Oxoglutaric acid	2 TMS	805.741	13.429	147
HMDB0000211	myo-Inositol	6 TMS	913.044	15.217	147
HMDB0000214	Ornithine	2 TMS	800.662	13.344	142
HMDB0000220	Palmitic acid	1 TMS	956.462	15.941	117
HMDB0000232	Quinolinic acid	2 TMS	881.575	14.693	147
HMDB0000239	Pyridoxine	3 TMS	883.862	14.731	147
HMDB0000251	Taurine	3 TMS	821.862	13.698	147
HMDB0000254	Succinic acid	2 TMS	642.862	10.714	147
HMDB0000258	Sucrose	8 TMS	1096.860	18.281	103
HMDB0000259	Serotonin	5 TMS	1127.110	18.785	174
HMDB0000262	Thymine	2 TMS	706.362	11.773	255
HMDB0000267	Pyroglutamic acid	2 TMS	376.732	6.279	73
HMDB0000271	Sarcosine	2 TMS	291.658	4.861	73
HMDB0000272	Phosphoserine	x TMS	857.062	14.284	147
HMDB0000283	D-Ribose	4 TMS	764.128	12.735	103
HMDB0000290	Uridine diphosphate-N-acetylglucosamine	n TMS	477.898	7.965	73
HMDB0000296	Uridine	3 TMS	1137.760	18.963	217
HMDB0000300	Uracil	2 TMS	1331.400	22.190	99
HMDB0000301	Urocanic acid	2 TMS	1019.080	16.985	147
HMDB0000303	Tryptamine	2 TMS	1067.710	17.795	174
HMDB0000306	Tyramine	3 TMS	903.612	15.060	174

HMDB0000491	3-Methyl-2-oxovaleric acid	3 TMS	562.928	9.382	89
HMDB0000500	4-Hydroxybenzoic acid	2 TMS	814.513	13.575	267
HMDB0000508	Ribitol	5 TMS	759.163	12.653	147
HMDB0000517	L-Arginine	3 TMS	873.263	14.554	157
HMDB0000574	L-Cysteine	4 TMS	1061.830	17.697	218
HMDB0000641	L-Glutamine	3 TMS	873.763	14.563	156
HMDB0000661	Glutaric acid	2 TMS	694.463	11.574	147
HMDB0000673	Linoleic acid	1 TMS	1019.560	16.993	95
HMDB0000687	L-Leucine	2 TMS	564.162	9.403	158
HMDB0000691	Malonic acid	2 TMS	568.813	9.480	147
HMDB0000695	Ketoleucine	3 TMS	558.913	9.315	89
HMDB0000696	L-Methionine	2 TMS	754.012	12.567	128
HMDB0000707	4-Hydroxyphenylpyruvic acid	3 TMS	432.898	7.215	73
HMDB0000719	L-Homoserine	3 TMS	671.262	11.188	128
HMDB0000725	4-Hydroxyproline	3 TMS	375.922	6.265	73
HMDB0000729	alpha-Hydroxyisobutyric acid	2 TMS	228.892	3.815	73
HMDB0000745	Homocarnosine	3 TMS	953.913	15.899	154
HMDB0000765	Mannitol	6 TMS	837.563	13.959	147
HMDB0000792	Sebacic acid	2 TMS	920.663	15.344	129
HMDB0000827	Stearic acid	1 TMS	1029.510	17.159	117
HMDB0000870	Histamine	3 TMS	928.313	15.472	86
HMDB0000883	L-Valine	2 TMS	522.763	8.713	144
HMDB0000898	1-Methylhistamine	2 TMS	918.963	15.316	174
HMDB0000929	L-Tryptophan	3 TMS	1073.360	17.889	202
HMDB0000930	trans-Cinnamic acid	1 TMS	815.990	13.600	131
HMDB0000954	Ferulic acid	2 TMS	1035.410	17.257	323
HMDB0000965	Hypotaurine	3 TMS	776.244	12.937	100
HMDB0000975	Trehalose	8 TMS	1135.280	18.921	147
HMDB0001123	2-Aminobenzoic acid	2 TMS	820.762	13.679	266
HMDB0001149	5-Aminolevulinic acid	3 TMS	840.713	14.012	174
HMDB0001256	Spermine	x TMS	1129.110	18.819	116
HMDB0001257	Spermidine	5 TMS	1919.820	31.997	73
HMDB0001262	Maltotriose	11 TMS	1338.930	22.316	204

HMDB0001336	3,4-Dihydroxybenzeneacetic acid	3 TMS	899.412	14.990	179
HMDB0001370	Diaminopimelic acid	4 TMS	915.463	15.258	200
HMDB0001388	alpha-Linolenic acid	1 TMS	1030.360	17.173	93
HMDB0001401	Glucose 6-phosphate	6 TMS	1038.610	17.310	147
HMDB0001406	Niacinamide	1 TMS	812.812	13.547	179
HMDB0001414	Putrescine	4 TMS	740.675	12.345	174
HMDB0001431	Pyridoxamine	3 TMS	470.287	7.838	73
HMDB0001488	Nicotinic acid	1 TMS	673.378	11.223	136
HMDB0001548	D-Ribose 5-phosphate	5 TMS	970.463	16.174	147
HMDB0001645	L-Norleucine	2 TMS	595.244	9.921	158
HMDB0001847	Caffeine	0 TMS	1063.860	17.731	109
HMDB0001851	L-Arabitol	5 TMS	762.013	12.700	103
HMDB0001856	Protocatechuic acid	3 TMS	886.991	14.783	193
HMDB0001893	alpha-Tocopherol	1 TMS	1347.710	22.462	237
HMDB0001901	Aminocaproic acid	3 TMS	808.562	13.476	174
HMDB0001925	Ibuprofen	1 TMS	827.975	13.800	160
HMDB0002064	N-Acetylputrescine	2 TMS	809.062	13.484	174
HMDB0002182	Phenylephrine	2 TMS	806.863	13.448	116
HMDB0002322	Cadaverine	4 TMS	815.628	13.594	174
HMDB0002329	Oxalic acid	2 TMS	528.013	8.800	147
HMDB0002641	2-Hydroxycinnamic acid	2 TMS	916.262	15.271	147
HMDB0002991	Cysteamine	3 TMS	1001.810	16.697	174
HMDB0003011	O-Acetylserine	2 TMS	681.743	11.362	174
HMDB0003070	Shikimic acid	4 TMS	850.613	14.177	204
HMDB0003431	L-Histidinol	3 TMS	915.025	15.250	154
HMDB0004041	L-Allothreonine	3 TMS	337.759	5.629	73
HMDB0011600	3-Methyladenine	1 TMS	1106.110	18.435	206
HMDB00042	Acetic acid	x TMS	351.600	5.860	117
HMDB0000237	Propionic acid	x TMS	439.200	7.320	131
HMDB0001873	Isobutyric acid	x TMS	451.800	7.530	145
HMDB0000039	Butyric acid	x TMS	475.200	7.920	145
HMDB0000718	Isovaleric acid	x TMS	481.800	8.030	159
HMDB0033742	2-Methylbutyric acid	x TMS	507.000	8.450	159

HMDB0000896	Valeric acid	x TMS	540.000	9.000	159
HMDB0033744	2-Methylpentanoic acid	x TMS	561.000	9.350	173
HMDB0033743	3-Methylpentanoic acid	x TMS	580.200	9.670	173
HMDB0034286	Isocaproic acid	x TMS	583.200	9.720	173
HMDB0000887	Caproic acid	x TMS	613.800	10.230	173
HMDB0033745	2-Methylhexanoic acid	x TMS	633.000	10.550	187
HMDB0033746	4-Methylhexanoic acid	x TMS	668.400	11.140	187
HMDB0000888	Heptanoic acid	x TMS	693.000	11.550	187

*****Study metabolites were filtered for quality control coefficient of variation (QC CV) values of less than 20% following batch correction using the ComBat method. This method corrects for batch effects in high-throughput biological data by applying an empirical Bayes framework to adjust for systematic variations across different batches. The use of the ComBat method ensures the reliability and reproducibility of the metabolite measurements by minimizing the potential impact of batch effects on the data, thus providing more accurate and consistent results.

******All metabolites were detected in positive electron ionization (EI) mode, which is a commonly used ionization technique in gas chromatography-mass spectrometry (GC-MS). Positive EI mode facilitates the generation of positively charged ions by bombarding the sample molecules with high-energy electrons, leading to ionization and fragmentation. This approach enhances the detection sensitivity and specificity for the metabolites analyzed.
